# The Rejuvenating Effect in Hot Asphalt Recycling by Mortar Transfer Ratio and Image Analysis

**DOI:** 10.3390/ma10060574

**Published:** 2017-05-24

**Authors:** Fusong Wang, Zipeng Wang, Chao Li, Yue Xiao, Shaopeng Wu, Pan Pan

**Affiliations:** 1 State Key Laboratory of Silicate Materials for Architectures, Wuhan University of Technology, Wuhan 430070, China; wangfs@whut.edn.cn (F.W.); lic@whut.edu.cn (C.L.); 2 Hebei Reach Traffic Engineering Consulting Co., Ltd., Shijiazhuang 050010, China; wangzipeng_sjz@126.com; 3 School of Resource and Civil Engineering, Wuhan Institute of Technology, Wuhan 430073, China; panp@wit.edu.cn

**Keywords:** rejuvenator, mortar transfer ratio, micro-cracks, preventive maintenance

## Abstract

Using a rejuvenator to improve the performance of asphalt pavement is an effective and economic way of hot asphalt recycling. This research analyzes the rejuvenating effect on aged asphalt by means of a Mortar Transfer Ratio (MTR) test, which concerns the ratio of asphalt mortar that moves from recycled aggregates (RAP aggregates) to fresh added aggregates when aged asphalt is treated with a regenerating agent and comes into contact with fresh aggregates. The proposed MTR test analyzes the regeneration in terms of the softening degree on aged asphalt when the rejuvenator is applied. The covered area ratio is studied with an image analyzing tool to understand the possibility of mortar transferring from RAP aggregates to fresh aggregates. Additionally, a micro-crack closure test is conducted and observed through a microscope. The repairing ability and diffusion characteristics of micro-cracks can therefore be analyzed. The test results demonstrate that the proposed mortar transfer ratio is a feasible way to evaluate rejuvenator diffusion during hot recycling. The mortar transfer ratio and uncovered area ratio on fresh aggregates are compatible, and can be used to quantify the contribution of the rejuvenator. Within a certain temperature range, the diffusing effect of the rejuvenator is better when the diffusing temperature is higher. The diffusion time of the rejuvenator is optimum when diffusion occurs for 4–8 h. When the rejuvenator is properly applied, the rough and cracking surface can be repaired, resulting in better covered aggregates. The micro-closure analysis visually indicates that rejuvenators can be used to repair the RAP aggregates during hot recycling.

## 1. Introduction

The field of road construction is a significant drain on manpower and material resources. It is expensive to excavate old materials and pave new materials again when bituminous pavement is contaminated [1,2,3]. During years’ worth of weathering and oxidation after construction, asphalt mixture deteriorates and therefore needs further maintenance or even total re-pavement [4,5,6].

Preventive maintenance means that before serious pavement distresses occur, using some preliminary methods to control pavement distresses and improve pavement performance will result in prolonging the service life of the pavement [7,8]. Many kinds of preventive maintenance methods have been used to protect asphalt pavement in recent years, including micro-surfacing, slurry seal, rejuvenator seal and chip seal [9]. Among these methods, rejuvenator seal, which is extended from the principle of the fog sealing method, is a preventive maintenance method for newly constructed pavement, specifically for use 3–4 years after construction [10,11]. The fog sealing method involves spraying fluid material, such as rejuvenator, emulsified asphalt and modified asphalt, creating a protective stratum on the pavement which fills the road cracks and stabilizes loose aggregates. 

Studies show that the service life of bituminous pavement can be prolonged by spraying rejuvenators in a regular and timely manner. The shortages of natural and human resources promote the development of research about the effect of rejuvenators on asphalt recycling, which has attracted the attention of many scholars [12,13,14].

However, the diffusion effect of the recycling agent in aged asphalt pavement is potentially problematic in application, because the interaction and diffusion between the rejuvenator and the aged asphalt are not only dependent on fluid mechanics, but also on molecular diffusion [15,16]. Moreover, not only does the regeneration effect of the recycling agent depend on physical and chemical properties of the regeneration agent itself, but it is also dependent on asphalt type, the degree of asphalt ageing, aggregate and filler types and other factors [17,18]. Hence, finding a highly-efficient method to determine rejuvenation is significant. Therefore, it is important to study the diffusion behavior of recycled asphalt mixture; it is of great scientific and economic significance [19]. 

The objective of this paper is to investigate the effectiveness of rejuvenation on aged asphalt by observing the process of mortar transfer during hot asphalt recycling and asphalt mortar micro-crack closure. The paper proposes an efficient method, the mortar transfer ratio (MTR) test, to evaluate the regeneration in terms of the degree of softening in aged asphalt and analyzes the suitable diffusion time and temperature. Meanwhile the uncovered area ratio that results from mortar transfer is analyzed by image software. By observing the micro-crack closure in asphalt mortar through a microscope, the crack repairing ability can be accurately analyzed. This research could be valuable to the engineering of asphalt pavement recycling technology and related scientific experiments in the future.

## 2. Experiment Materials

**Figure 1:**
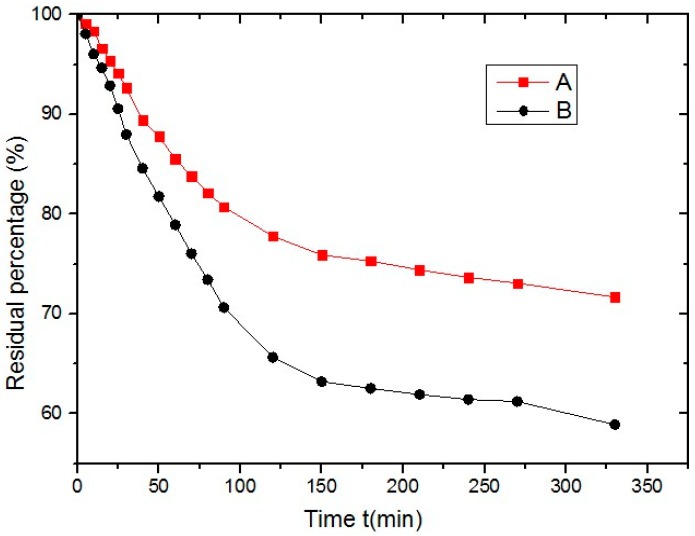
Natural evaporation curves of two used rejuvenators at 60 °C.

Andesite aggregate and pen 90 grade heavy traffic asphalt binder (AH90) were used as raw materials. Table 1 shows the fundamental characteristics of the asphalt binder used in the experiment. Test methods listed in Table 1 refer to Chinese official standard JTG E20-2011 [20], which are the standard test methods of asphalt and asphalt mixtures for Highway Engineering. Moreover, two types of rejuvenators (A and B) were utilized in the experiment, the technical parameters of which are schematized in Table 2. Figure 1 compares the volatile speed and residue amount. Rejuvenator-A had bigger viscosity and more solute components than that of rejuvenator-B. Consequently, the residual percent of rejuvenator-A was much higher than that of rejuvenator-B, which means more soft and active content exists in rejuvenator-B than in rejuvenator-A.

## 3. Experimental Methods

### 3.1. Mortar Transfer Ratio

In the hot-in-place recycling process, the scarified aged asphalt mixture is first heated and remixed. Then the required amount of fresh aggregates and binder (fresh asphalt binder and rejuvenator) is added. Fresh aggregates are added to rebuild the aggregate gradation to ensure sufficient loading capacity, while additional binder is added to soften the aged binder and provide better viscoelastic behavior [21,22]. 

**Figure 2:**
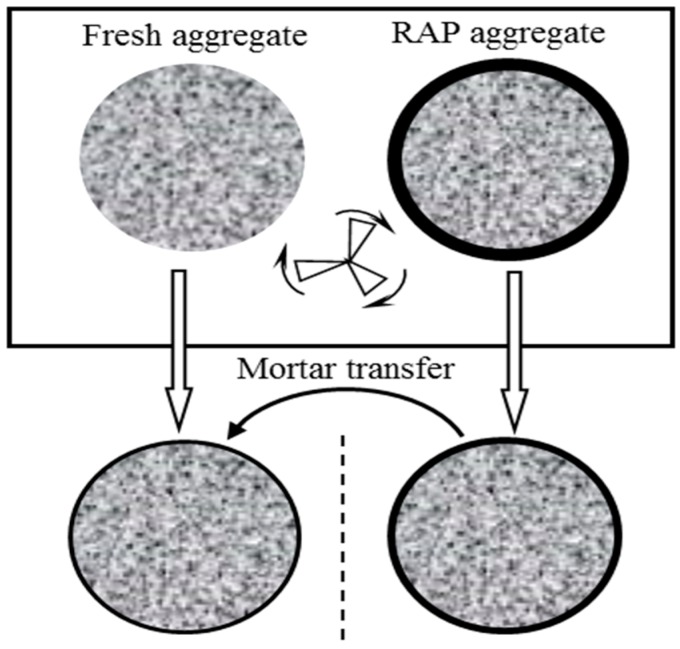
Schematic diagram of mortar transfer ratio during hot recycling.

In this research, the mortar transferred from aged aggregates (RAP aggregates) to fresh aggregates during the hot recycling process was characterized by mortar transfer ratio. A simple but effective test program was proposed to study the mortar transfer ratio, which is described in Figure 2. 

The mortar transfer test was conducted as follows: firstly, clean and dried aggregates with a grain size between 10–20 mm were mixed with asphalt binder. Then, this mixture was placed in an oven for accelerated ageing purposes at 120 °C for 5 days. At the end of that time, those aggregates which were covered with aged binder, so called as RAP aggregates, were obtained. Although these aggregates with aged binder are different from real RAP, they were used to represent real RAP because the mortar weight cannot be defined with real RAP.

After accelerated ageing, RAP aggregates were remixed with fresh aggregates with a grain size between 20–30 mm for another 90 s. Mortar transfer ratio (MTR) can be calculated with Equation (1). The difference of grain size between RAP aggregates and fresh aggregates was set for easier distinguishing in the transferred mortar.

(1) $$T_{R}=\frac{W_{r}-W_{f}}{W_{m}-W_{a}}\times 100\%$$

where, *W_a_* is the weight of clean and dried aggregates; *W_m_* is the weight of RAP aggregates which is equal to *W_a_* plus aged binder on the aggregates surface; *W_f_* is the weight of added fresh aggregates; *W_r_* is the weight of added fresh aggregates covered with transferred aged binder.

Before mixing the RAP aggregates with fresh aggregates, the rejuvenator was uniformly sprayed to the RAP aggregates’ surface to study the diffusion and rejuvenation effect. Diffusion at 20 °C, 35 °C, 50 °C for 2 h, 4 h, 8 h and 24 h were compared and analyzed. The higher the mortar transfer ratio was, the better the effect of the regeneration was during recycling.

Imana, an image stripping analysis program that was developed by Highway Research Lab at Kunsan National University [23], was used to further analyze the coverage of mortar on fresh aggregates. Imana shows the asphalt covered area and uncovered area accurately, indicating to what extent the studied rejuvenator softened the aged mortar and enhances the transferred mortar from RAP aggregates to fresh aggregates. A higher stripping area means a lower covered area, indicating worse mortar transfer [24].

### 3.2. Micro-Crack Closure

**Figure 3:**
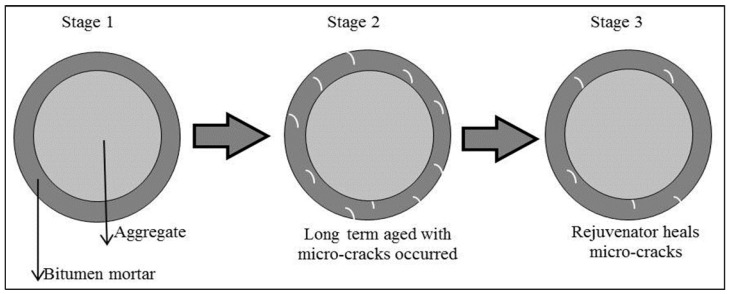
Schematic diagram of asphalt mortar micro-crack closure test.

Ageing will cause the asphalt binder to lose its flexibility and hence, result in micro-cracks. Such micro-cracks can decrease the bonding strength between aggregates, causing pavement cracking, potholes or raveling [25]. Rejuvenators were proposed to be used in hot recycling to repair those micro-cracks [26]. A micro-crack closure analysis was suggested to address the contribution of using rejuvenators in hot recycling. Figure 3 explains the micro-crack closure test.

The test was conducted as follows: firstly, aggregates covered in 1 mm thick of asphalt mortar were prepared by mixing aggregates with asphalt mortar, which contained 80% of andesite filler and 20% of asphalt binder. The mortar thickness was ensured by checking the amount of covered mortar and the total surface area of used aggregates. Then the covered aggregates were placed in an oven for accelerated ageing purposes at 120 °C for 5 days. At the end of that time, features of micro-cracks on the surface of RAP aggregates and rejuvenator-treated aggregates were assessed and comparatively analyzed. A microscope was used here for micro-crack analysis. 

Accounting for the diffusing rate, two days’ diffusion—which leads to fully curing and therefore can minimize the influence of diffusing time—was conducted before microscope analysis. The rejuvenator treated aggregates were divided into three groups of diffusion at 20 °C, 50 °C and 150 °C for two days. 

## 4. Results Analysis

### 4.1. Mortar Transfer Ratio

At the beginning of this research, MTR analysis was conducted by the process of blending RAP and fresh aggregates, without applying any rejuvenators. The experiment results were used as reference value for studying the effect of using rejuvenators. Without rejuvenators, the mortar transfer ratio from RAP aggregates to fresh aggregates is 2.93%, which is only 2.198 g out of 75 g of binder transferred onto the fresh aggregates. 

**Figure 4:**
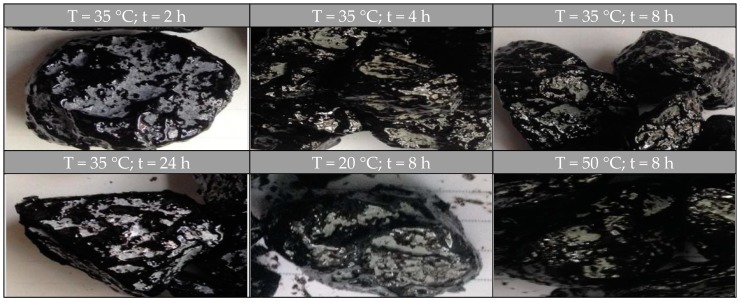
Fresh aggregates covered with rejuvenator-A treated binder.

Figure 4 shows the individual fresh aggregates covered with transferred binder from the RAP aggregates’ surface when rejuvenator-A was applied. When the diffusion temperature or reacting time increases, there is a significant increase in the covered area on the fresh aggregates that can be observed. 

The MTR results for rejuvenator-A were calculated using Equation (1) and presented in Table 3. It can be clearly observed that 10.67 was the maximum MTR value, which happened at 35 °C for 4 h. This means 4 h diffusion at 35 °C resulted in the best softening effect on the aged binder. Further diffusion did not have an effect on increasing MTR values. Meanwhile, 4.29 was the minimum MTR value, which was obtained at 20 °C for 8 h. According to Table 3, the MTR values doubled from 20 °C (8 h) to 35 °C (8 h), and then slightly decreased from 35 °C (8 h) to 50 °C (8 h). It is logical to conclude that the rejuvenator diffused faster at 35 °C than at 20 °C, because of the temperature sensitivity of the bitumen binder. At higher temperatures, such as 50 °C, the covered binder might become too soft to get transferred to fresh aggregate, and hence, result in slightly lower MTR. Furthermore, when the grain number of fresh aggregates increases, it can also increase the chances of hot-mixed asphalt transfer to the fresh aggregates, resulting in a larger mortar transfer ratio. This is because with the same aggregate weight, the higher the number of grains the aggregates have, the bigger the surface area will be that comes into contact with RAP aggregates.

**Figure 5:**
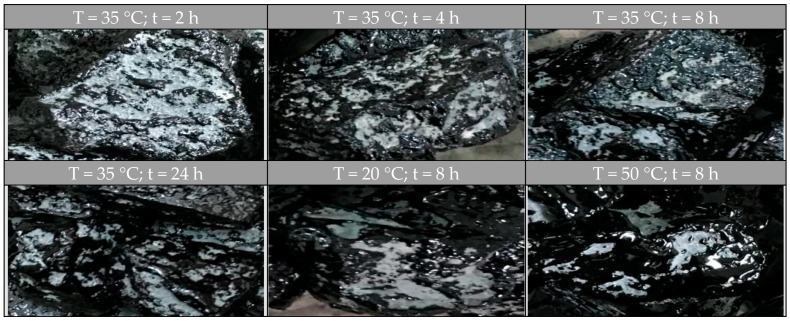
Fresh aggregates covered with rejuvenator-B treated binder.

Figure 5 shows the individual fresh aggregates covered with transferred binder from RAP aggregates’ surface when rejuvenator-B was applied. Once again, the images indicate that diffusion temperature or reacting time has positive effect on MTR characteristics.

Table 4 illustrates the MTR of the rejuvenator-B treated specimens. It indicates that rejuvenator-B has a positive effect in curing aged asphalt. In addition, 11.87 is the maximum with the condition of 50 °C and 8 h for the mortar transfer ratio and 8.93 is the minimum with the condition of 35 °C and 4 h. And it can be concluded that lower temperature conditions result in a worse diffusion effect.

Obviously, the big increase on MTR values between no rejuvenator-treated and rejuvenator-treated specimen demonstrates that the two investigated rejuvenators cause effective regeneration on aged asphalt. 

**Figure 6:**
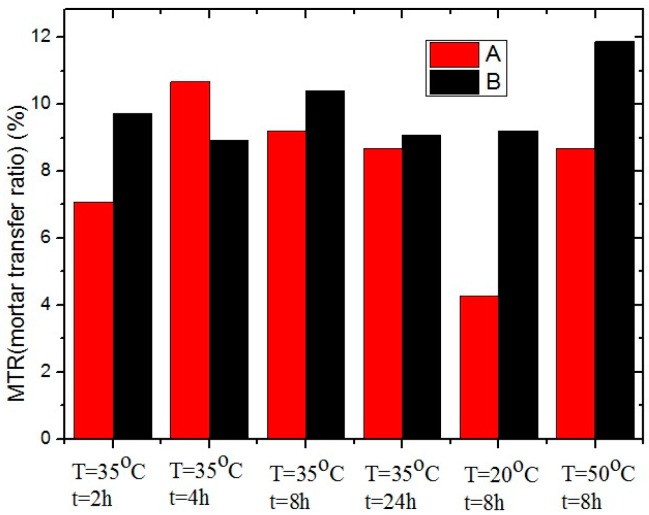
Histogram of mortar transfer ratio for rejuvenator-A and rejuvenator-B.

Figure 6 compares the rejuvenation effects between rejuvenator-A and rejuvenator-B by means of MTR values. The results show that rejuvenator-B has a better diffusion effect than rejuvenator-A, because the MTR values of rejuvenator-B treated specimens are consequently higher than that of rejuvenator-A treated specimens. Furthermore, in a certain temperature range, the diffusion effect of the rejuvenator is better when the diffusing temperature is higher. Moreover, 4–8 h of diffusion is already sufficient for optimal diffusion effect.

### 4.2. Image Analysis

**Figure 7:**
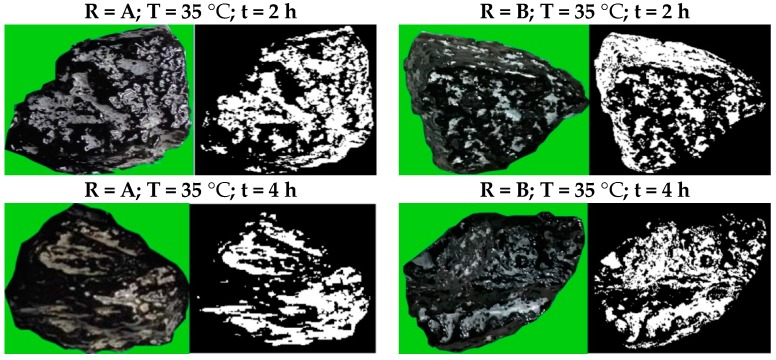

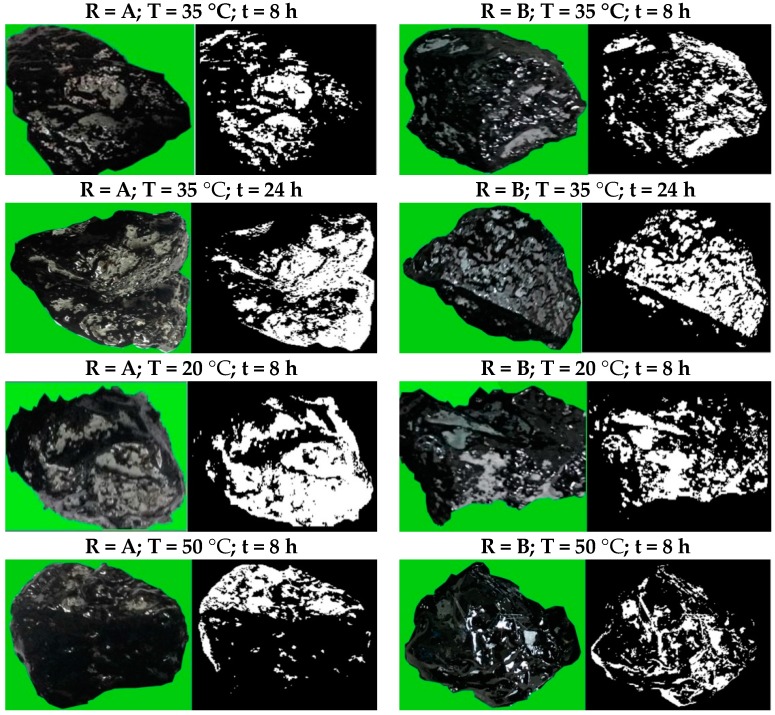
Image analysis in mortar transfer ratio (MTR) test.

Figure 7 presents the analyzed surface areas of fresh aggregates that were covered with transferred binder. The corresponding uncovered area ratios were concluded in Table 5. The Imana program is designed to characterize the stripping ratio to study the moisture resistance in the asphalt mixture. Technically, the stripping ratio means the asphalt mortar is stripped from aggregates during moisture damage. Therefore, a smaller stripping area ratio reflects a bigger covered surface area. In this research, the smaller stripping area ratio illustrates less uncovered surface, indicating more transferred binder from RAP aggregates to fresh aggregates. It therefore means a better regenerative effect from the rejuvenator. 

It can be concluded from Table 5 that the smallest uncovered area ratio was generated when the experimental condition was 50 °C and diffusing time was 8 h, both in rejuvenator-A treated and rejuvenator-B treated specimens. Furthermore, the biggest uncovered area ratio occurred when rejuvenator-A diffused at 20 °C for 8 h; while rejuvenator-B generated the maximum stripping uncovered area ratio at 35 °C for 2 h. A similar phenomenon was noticed with the mortar transfer ratio where it was found that a suitable diffusing time is between 4–8 h. A higher diffusion temperature may generate a smaller uncovered area, which means a better rejuvenator effect. 

In addition, it is difficult to describe which rejuvenator presented better regeneration contributions by means of image analysis. Rejuvenator-A presented a similar uncovered area ratio in a same condition with B, but had stronger thermostability than B under different conditions.

### 4.3. Micro-Crack Closure

**Figure 8:**
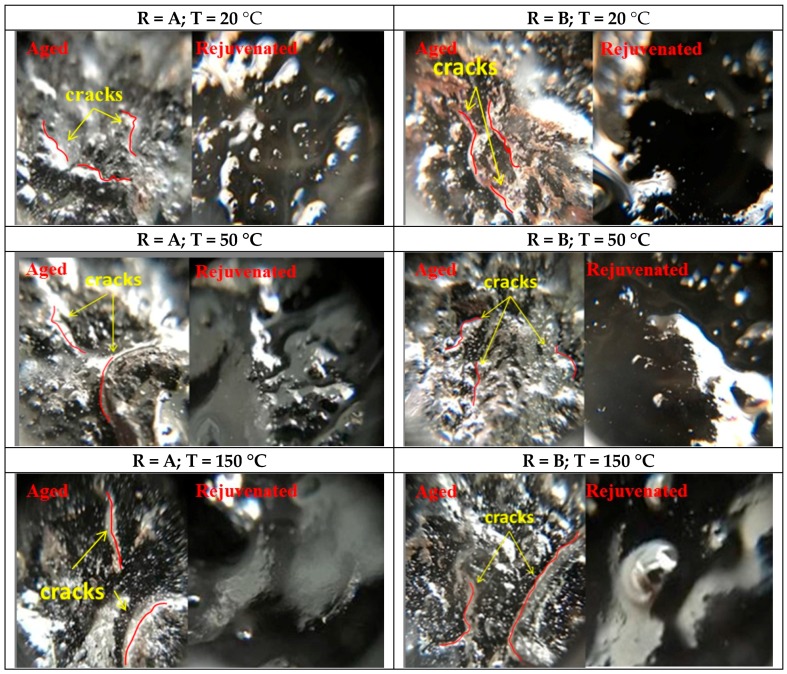
Micro-crack closure in asphalt mortar samples.

Micro-cracks can be observed in asphalt mortar on aggregate surface after serious ageing. After spraying the rejuvenators, Figure 8 (“R” means rejuvenator) depicts the micro-cracks with different diffusing temperatures and rejuvenators which were observed from the aggregate surface with a microscope. The magnification was 60 times. Diffusion time in this test was fixed at 48 h, and the spraying amount of rejuvenator was 10 g each group.

As evident, cracks widely exist on the surface of RAP aggregates. When the rejuvenator was properly applied, the rough and cracking surface was repaired, resulting in better covered aggregates. The micro-closure analysis visually demonstrates that rejuvenators can be used to repair the RAP aggregates during hot recycling. Moreover, better micro-crack effect can be achieved at a higher conditioning temperature.

## 5. Conclusions

Mortar transfer ratio and micro-closure analysis were proposed in this research to investigate the diffusion effect of rejuvenators when they are used during hot recycling of asphalt mixtures. Based on the research results, the following conclusions can be drawn:

(1) The results of the experiment demonstrate that the mortar transfer ratio test of asphalt is a feasible way to evaluate the rejuvenator diffusion during hot recycling. The mortar transfer ratio and uncovered area ratio on fresh aggregates are compatible, and can be used to quantify the rejuvenator contribution.
(2) Within in a certain temperature range, the diffusion effect of the rejuvenator is better when the diffusion temperature is higher. Four to eight hours of diffusing time is already enough for optimal diffusion effect.
(3) When the rejuvenator was properly applied, the rough and cracking surface was repaired, resulting in better covered aggregates. The micro-closure analysis visually demonstrates that rejuvenators can be used to repair the RAP aggregates during hot recycling.

Further research is required to improve the applicability for the assessment of the effect of rejuvenators in the field. For example, the time of rejuvenation should be discussed in more detail in MTR testing; additional types of binder and aggregates should be included to verify the MTR test; and the final performance of the rejuvenator-treated asphalt mixture should also be investigated. 

## Figures and Tables

**Table 1:** Characteristics of the used asphalt binder.

Properties	Values	Specifications	Test Method
Penetration (25 °C) [0.1 mm]	85	80–100	T0604-2011
Softening point [°C]	44	≥44	T0604-2011
Ductility, 5 cm/min, 15 °C [cm]	>100	>100	T0605-2011
Wax content [wt %]	0.83	≤3.0	T0615-2011
Flash point (COC) [°C]	300	≥245	T0611-2011
Solubility (C_2_HCl_3_) [wt %]	99.7	≥99.5	T0607-2011

**Table 2:** Technical parameters of rejuvenators.

Properties	Rejuvenator A	Rejuvenator B	Test Method
Form	Liquid	Liquid	
Color	pink	Brown	
pH	4.8	5.6	pH test paper
Viscosity (25 °C), SFS	40	15	ASTM D-244
Solute component [wt %]	65	60	ASTM D-244
Regeneration component [wt %]	8	8	ASTM D-2006-70
Asphaltene component [wt %]	0.4	0.75	

**Table 3:** MTR results of rejuvenator-A treated specimens.

*W_a_* [kg]	*W_m_* [kg]	T [°C]	t [h]	Fresh Aggregates			*T_R_* [%]
				*W_f_* [g]	*W_r_* [g]	Grain Number	
1.3322	1.4072	35	2	498.9	504.2	17	7.07
1.3306	1.4056	35	4	501.1	509.1	18	10.67
1.3232	1.3982	35	8	499.4	506.5	19	9.20
1.3246	1.3996	35	24	496.1	502.6	19	8.67
1.3356	1.4106	20	8	498.2	501.4	19	4.29
1.3356	1.4106	50	8	498.5	505.0	19	8.67

**Table 4:** MTR results of rejuvenator-B treated specimens.

*W_a_* [kg]	*W_m_* [kg]	T [°C]	t [h]	Fresh Aggregates			*T_R_* [%]
				*W_f_* [g]	*W_r_* [g]	Grain Number	
1.3382	1.4132	35	2	479.6	486.9	19	9.73
1.3402	1.4152	35	4	494.2	500.9	19	8.93
1.3466	1.4216	35	8	480.6	488.4	19	10.40
1.3321	1.4071	35	24	414.1	420.9	19	9.07
1.3512	1.4262	20	8	407.3	414.2	19	9.20
1.3545	1.4295	50	8	493.3	502.2	19	11.87

**Table 5:** Uncovered area ratio from image analysis.

Test Condition	Uncovered Ratio for A Treated Specimen (%)	Uncovered Ratio for B Treated Specimen (%)
**T = 35 °C; t = 2 h**	47.31	45.20
**T = 35 °C; t = 4 h**	39.30	40.80
**T = 35 °C; t = 8 h**	28.24	32.98
**T = 35 °C; t = 24 h**	48.46	46.57
**T = 20 °C; t = 8 h**	57.74	36.78
**T = 50 °C; t = 8 h**	23.13	26.53
